# Supplementary material for: Nitric oxide radicals are emitted by wasp eggs to kill mold fungi
Source: eLife. 2019 Jun 11;8:e43718. doi: 10.7554/eLife.43718 (PMC6559793; doi:10.7554/eLife.43718)

Strohm et al.
Figure 4 Source data
Eggs injected with DAR4M-AM

The columns are ordered as follows:

1h, 3h, 5h, 24h, 72h after injection.

Note: for some eggs not all time points are available (in particular for 72h after injection).

Page 1: *Philanthus triangulum* eggs injected with Phosphate buffer; no 5h images available

Page 2-7: *Philanthus triangulum* eggs injected with DAR4M-AM in phosphate buffer;

eggs damaged by handling (mechanical damage, desiccation of the storage container) during incubation are indicated by an asterix

Page 8: *Philanthus triangulum* eggs injected with DAR4M-AM in phosphate buffer

Page 9: *Ampulex compressa* eggs injected with DAR4M-AM in phosphate buffer

Page 10-11: *Osmia bicornis* eggs injected with DAR4M-AM in phosphate buffer; peripheral strong fluorescence is caused by adhering pollen

Ianthus triangulum – Control eggs

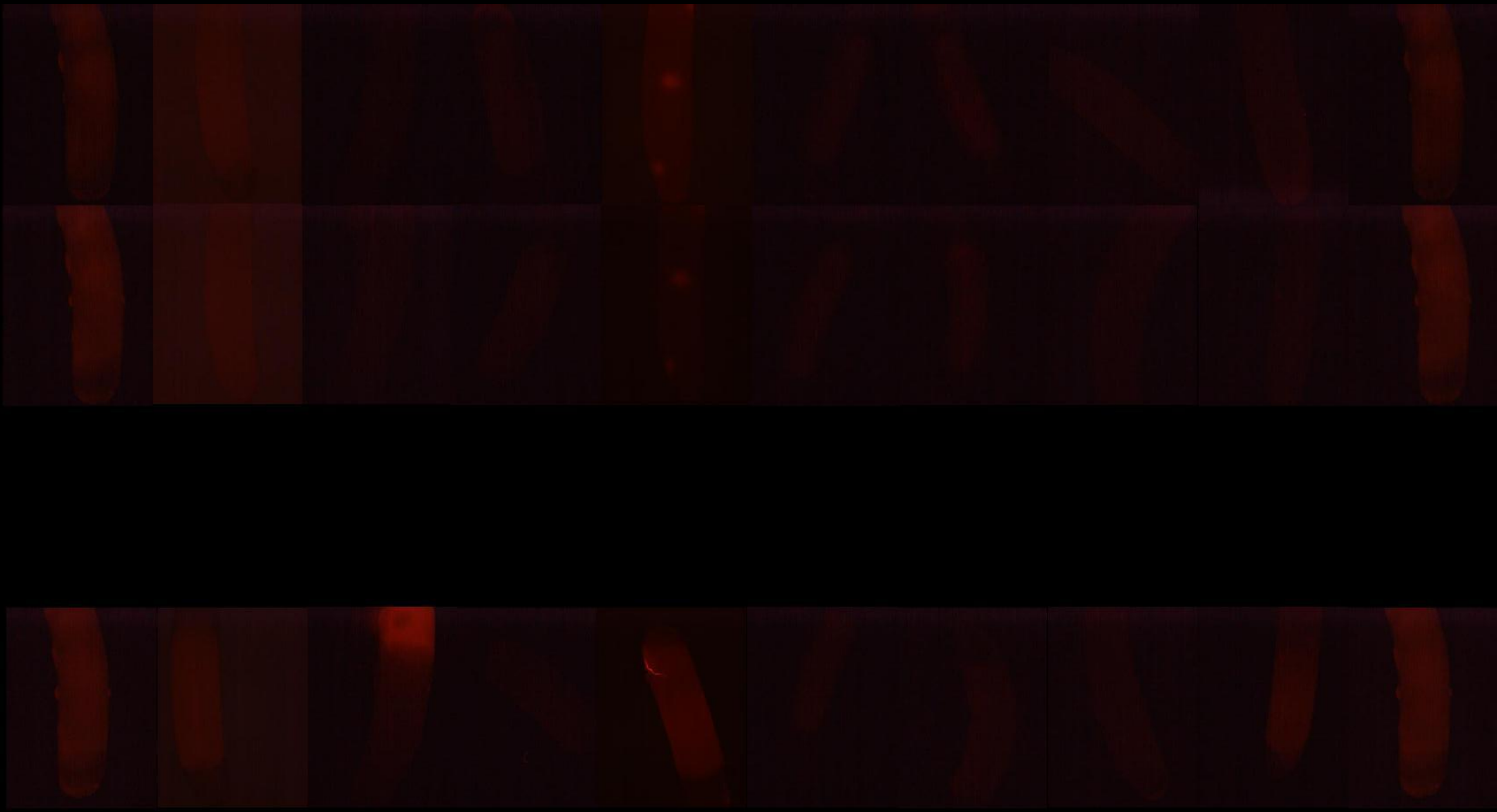

Philanthus triangulum – DAR4M-AM injected eggs

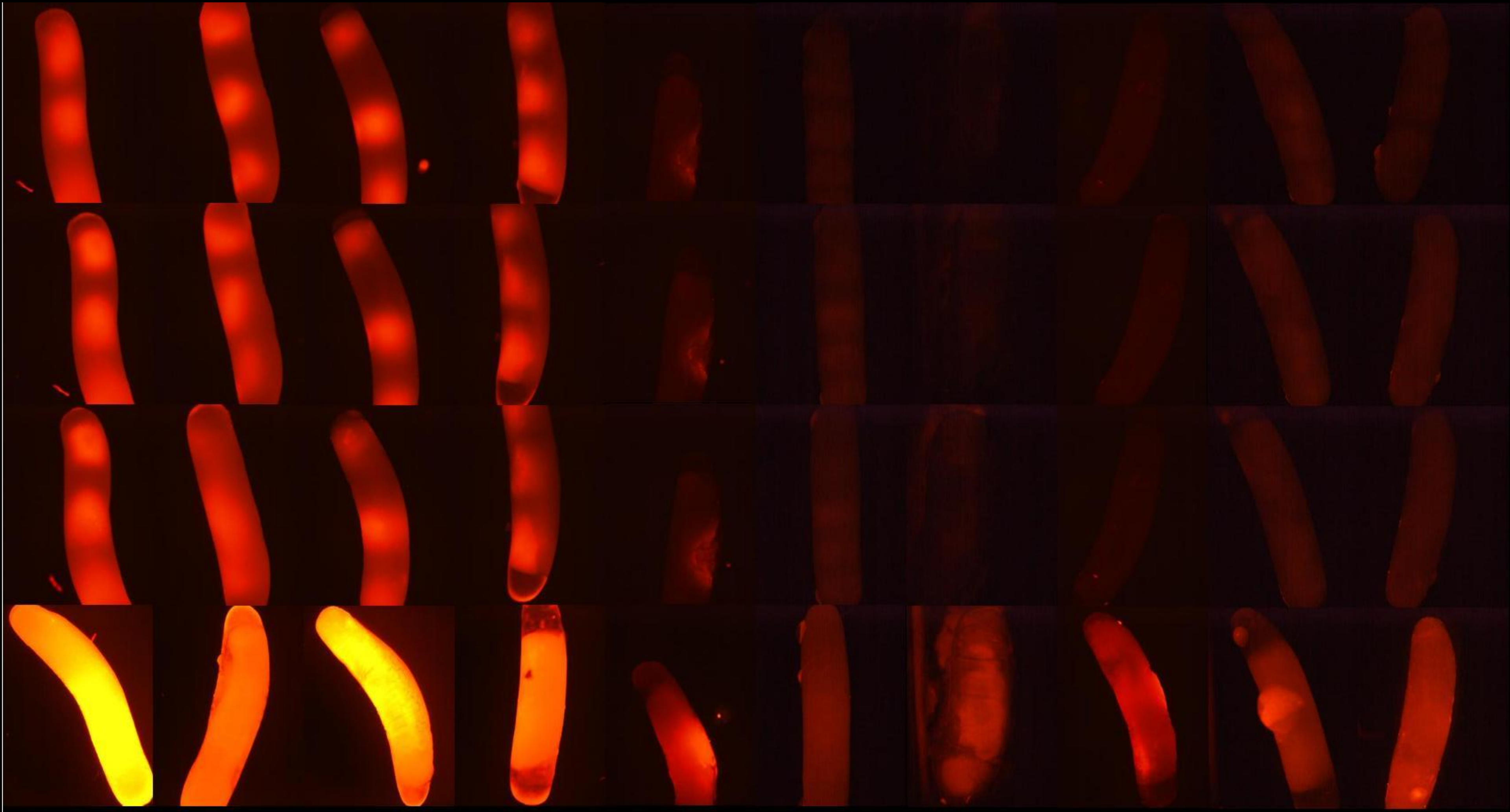

Philanthus triangulum – DAR4M-AM injected eggs

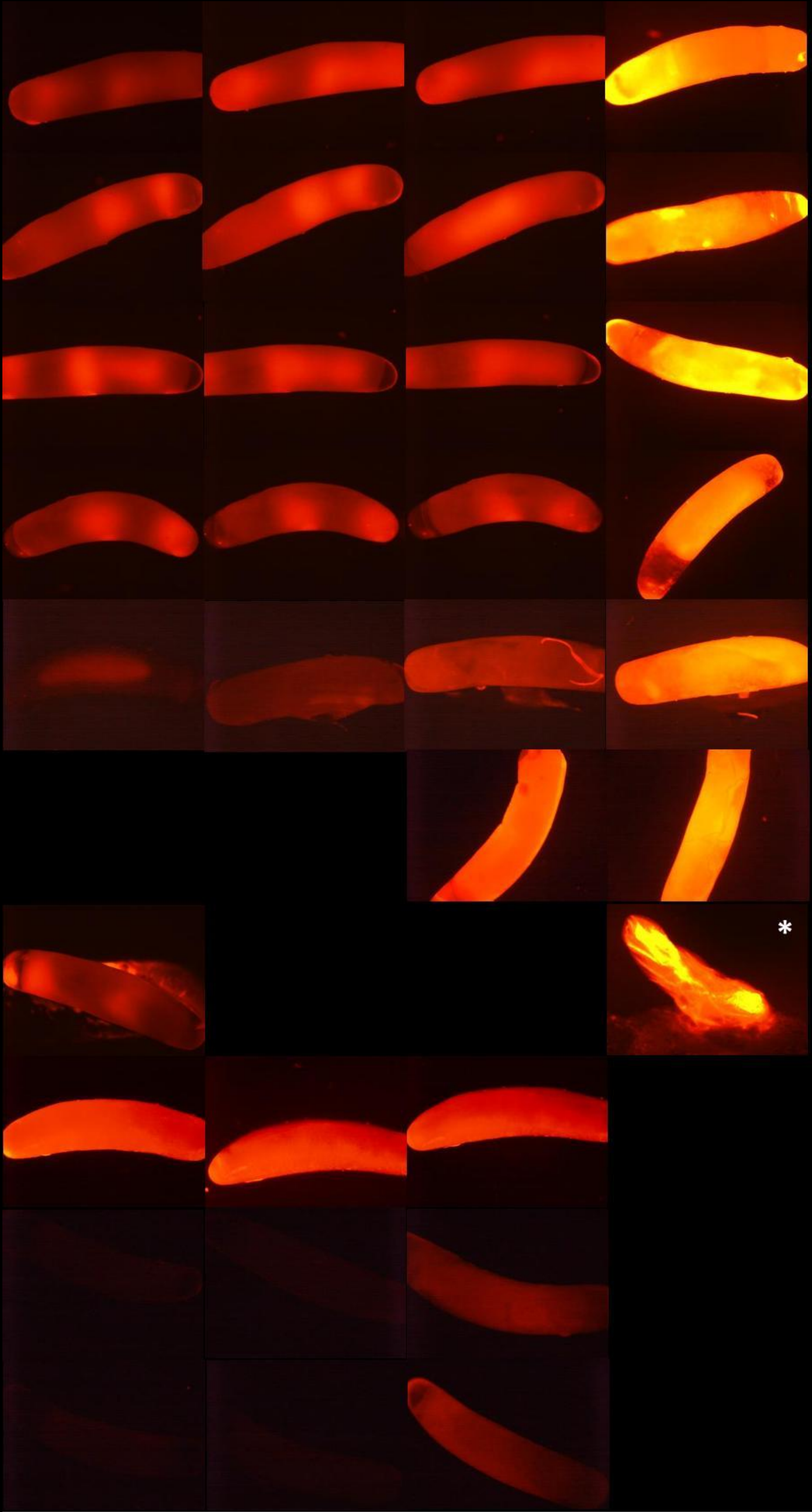

Philanthus triangulum – DAR4M-AM injected eggs

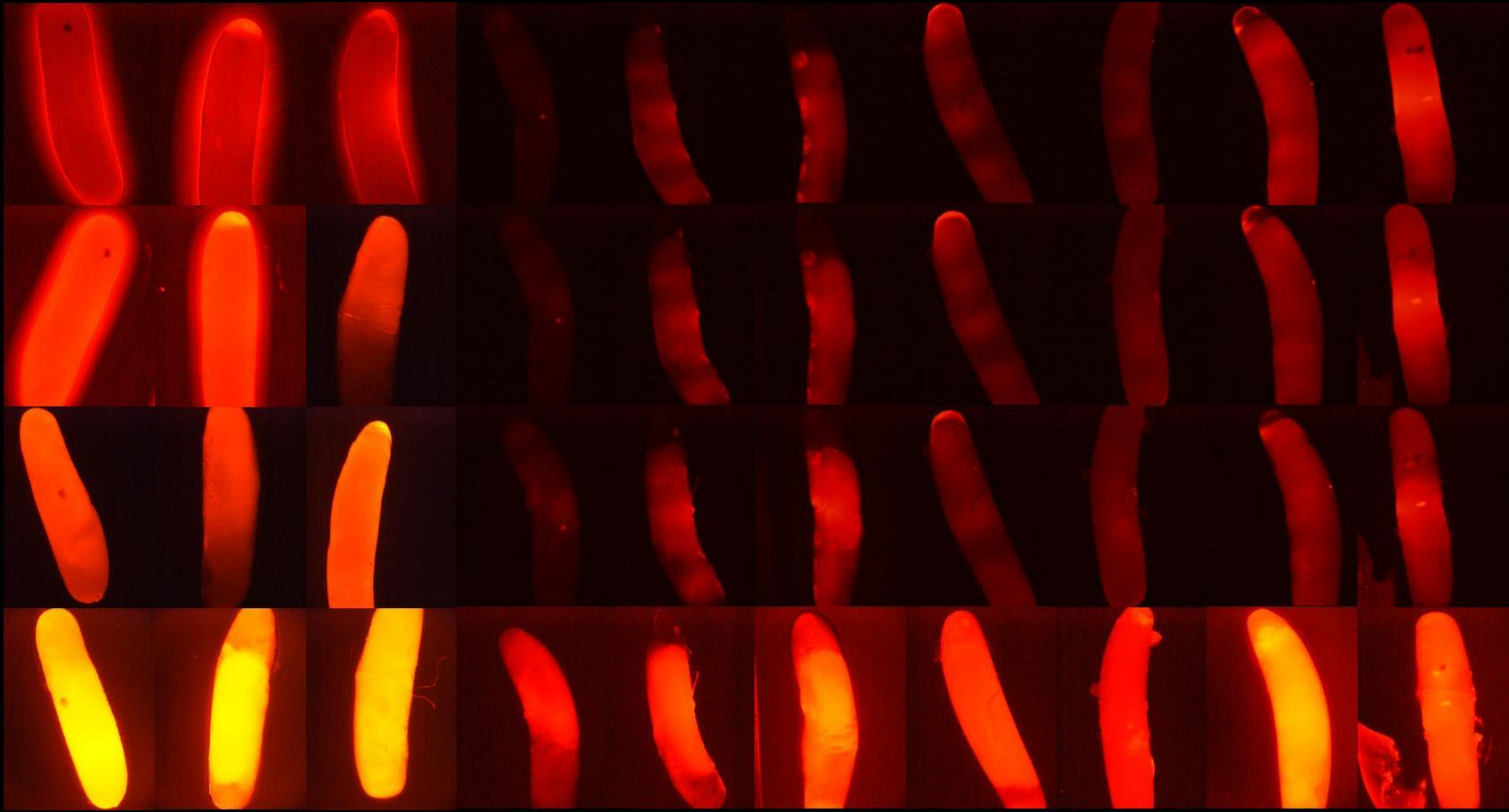

Philanthus triangulum – DAR4M-AM injected eggs

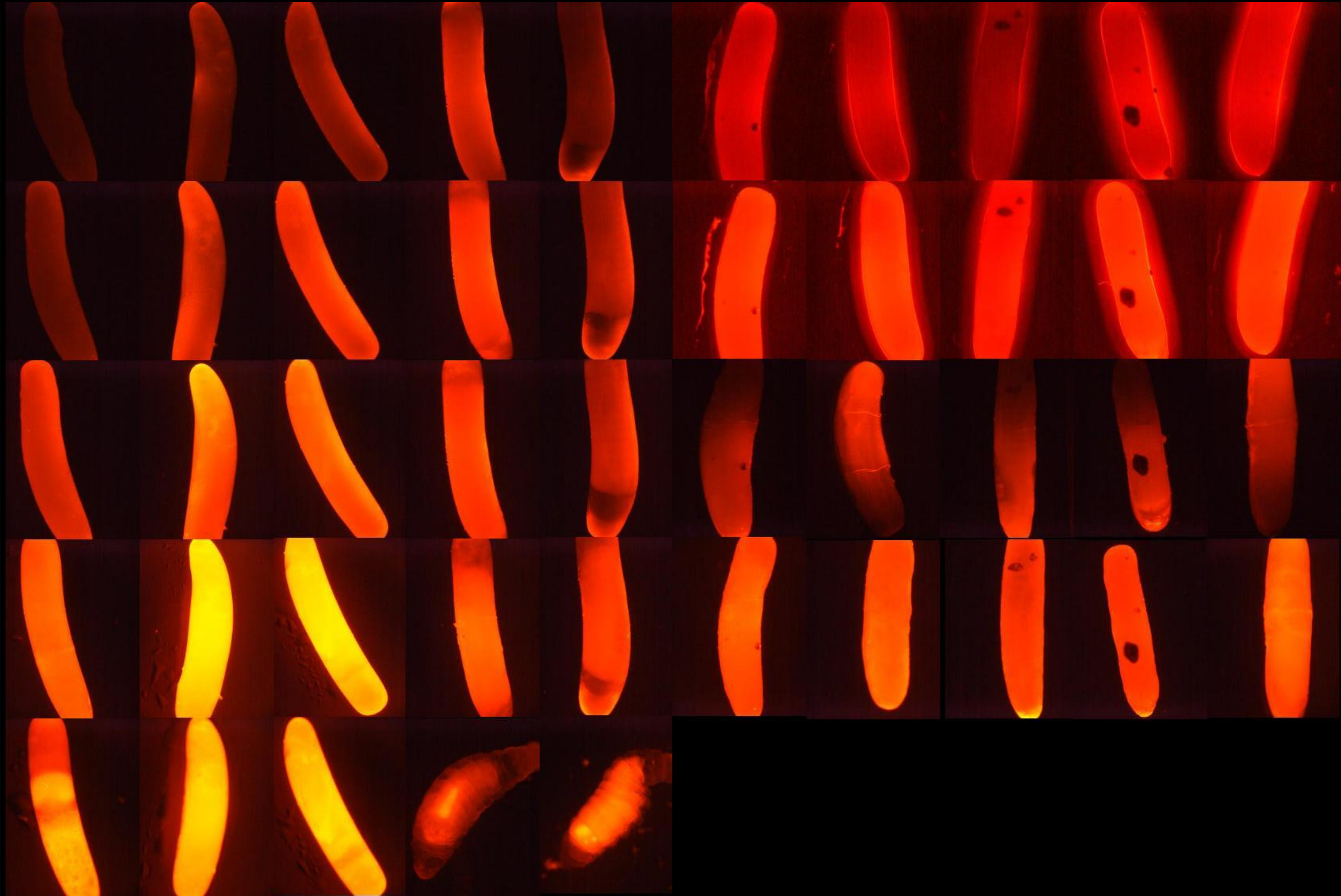

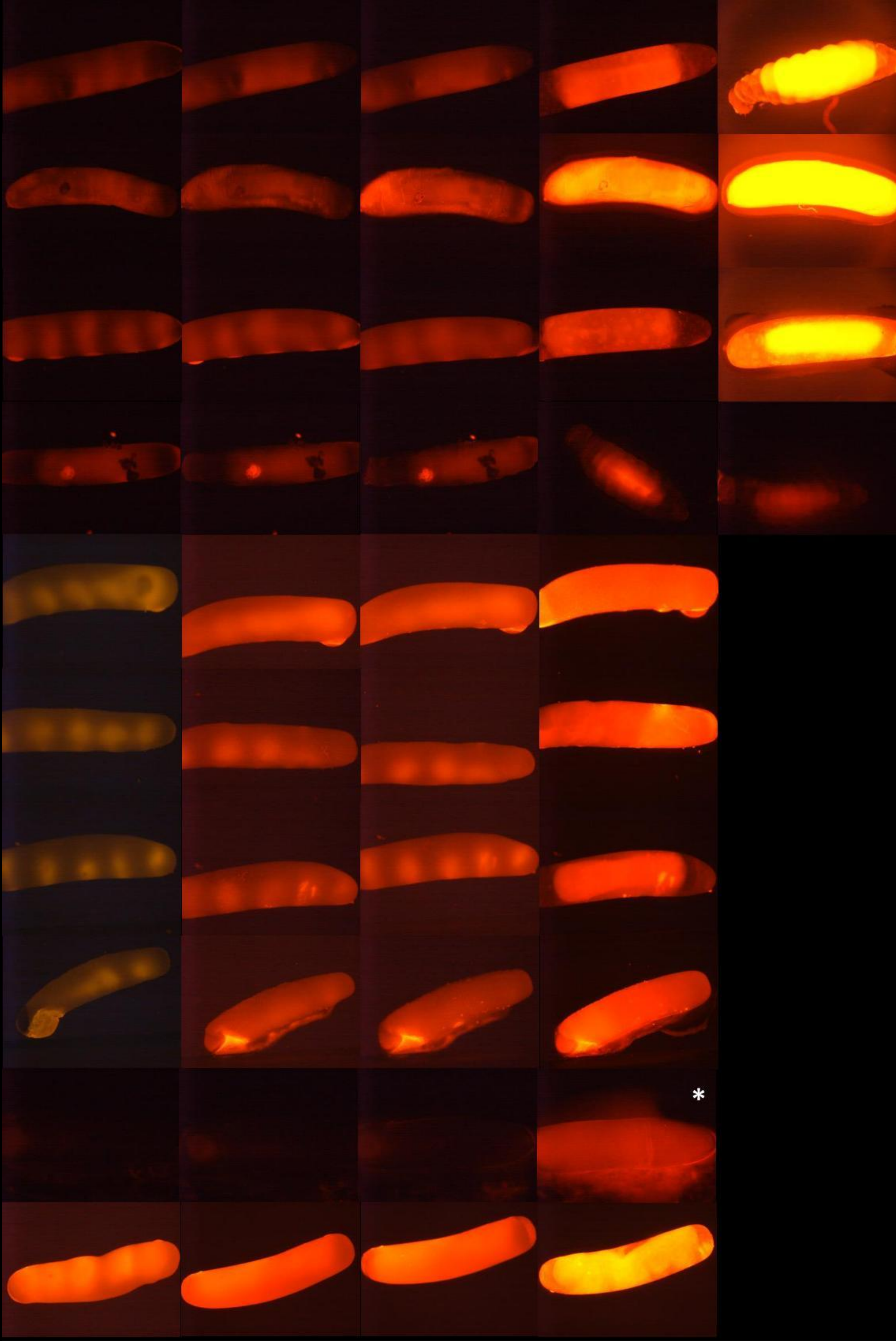

Philanthus triangulum – DAR4M-AM injected eggs

Philanthus triangulum – DAR4M-AM injected eggs

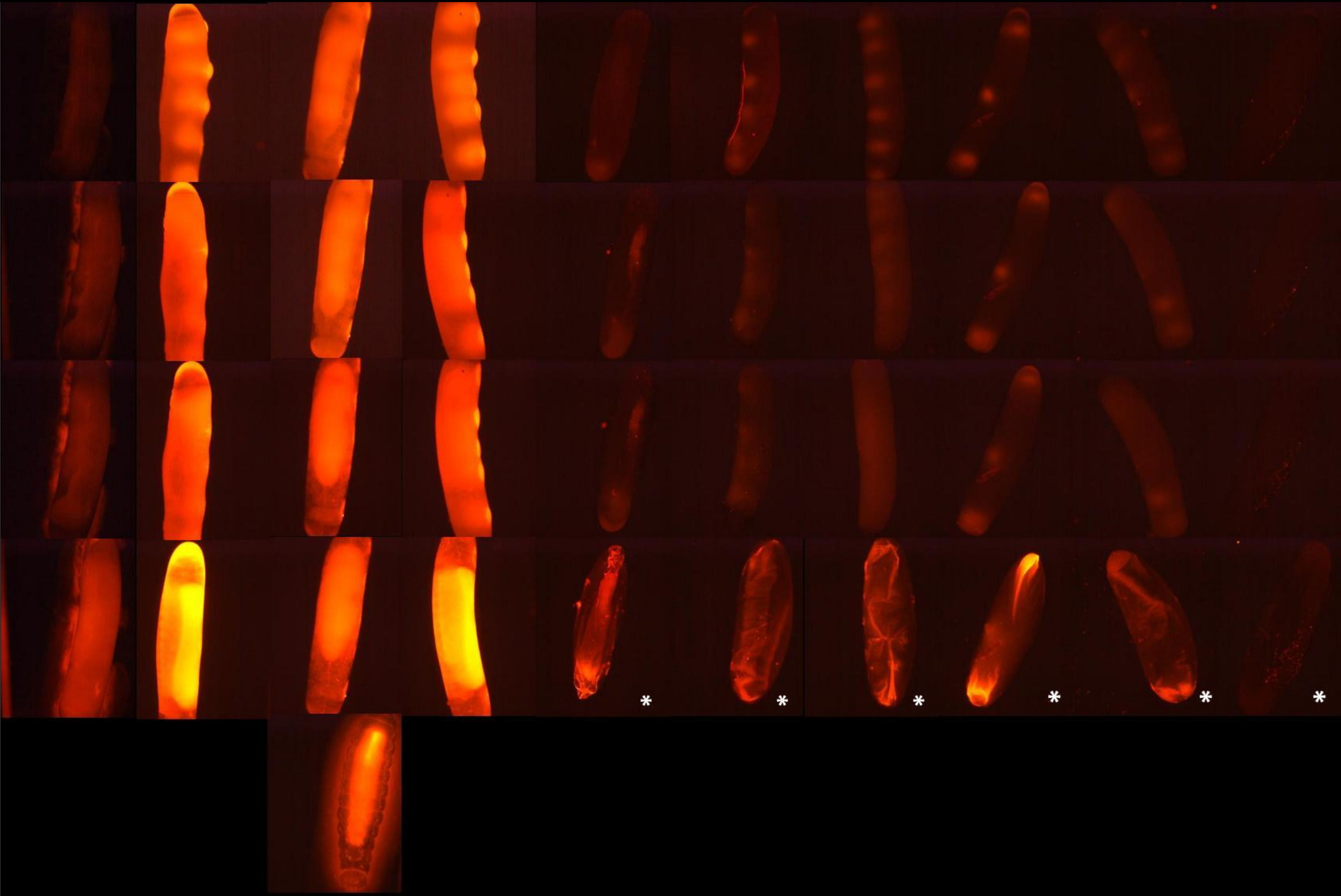

Philanthus triangulum – DAR4M-AM injected eggs

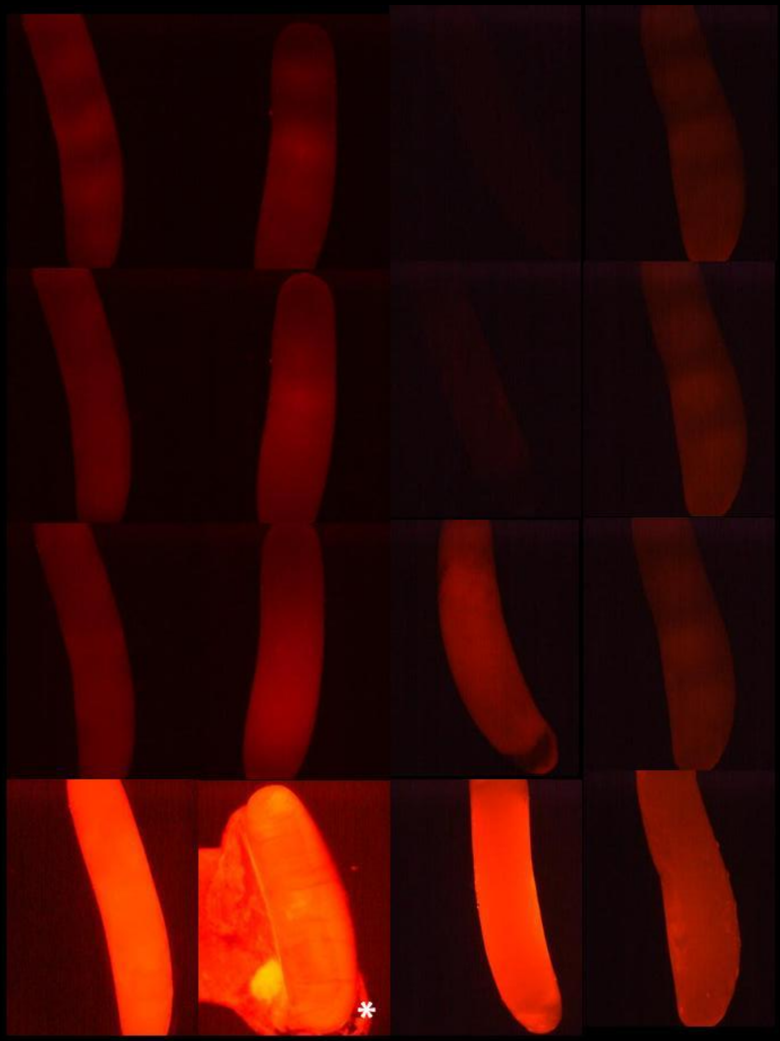

Philanthus triangulum – DAR4M-AM injected larvae

Beewolf – DAR4M-AM injected

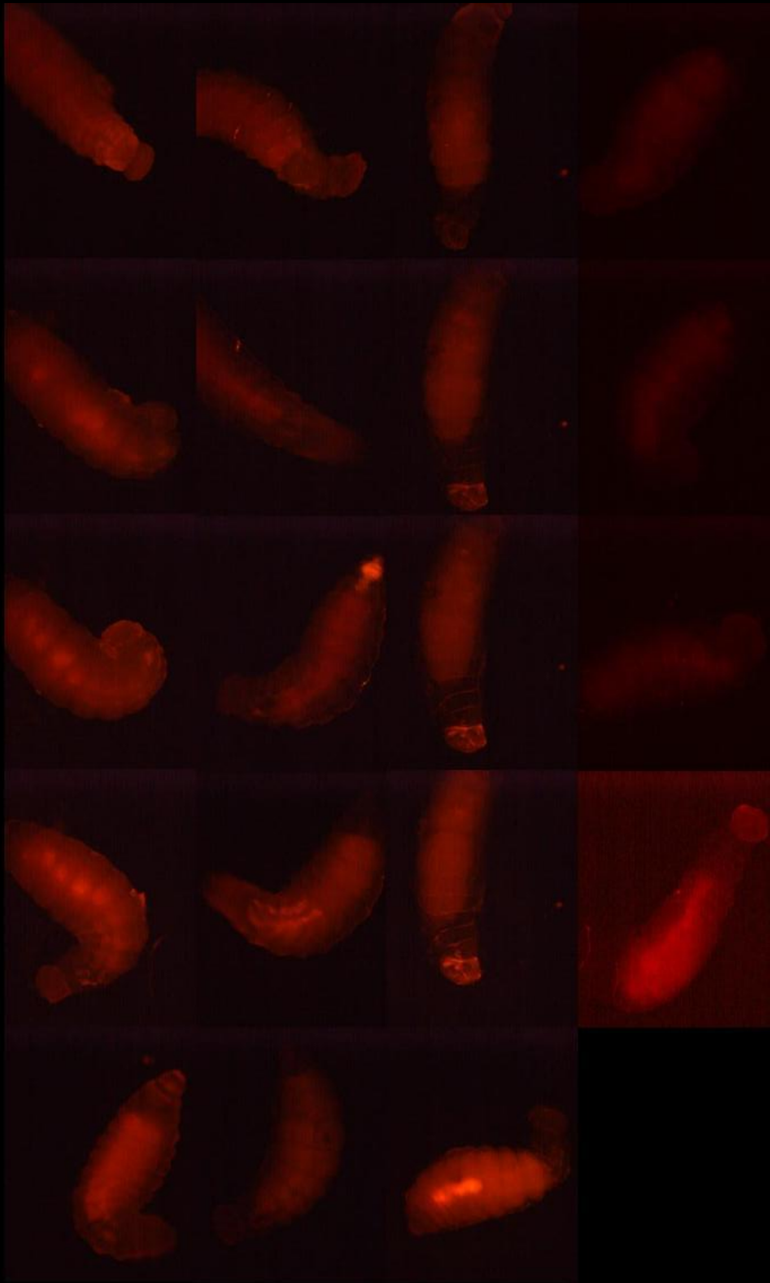

Ampulex compressa – DAR4M-AM injected eggs

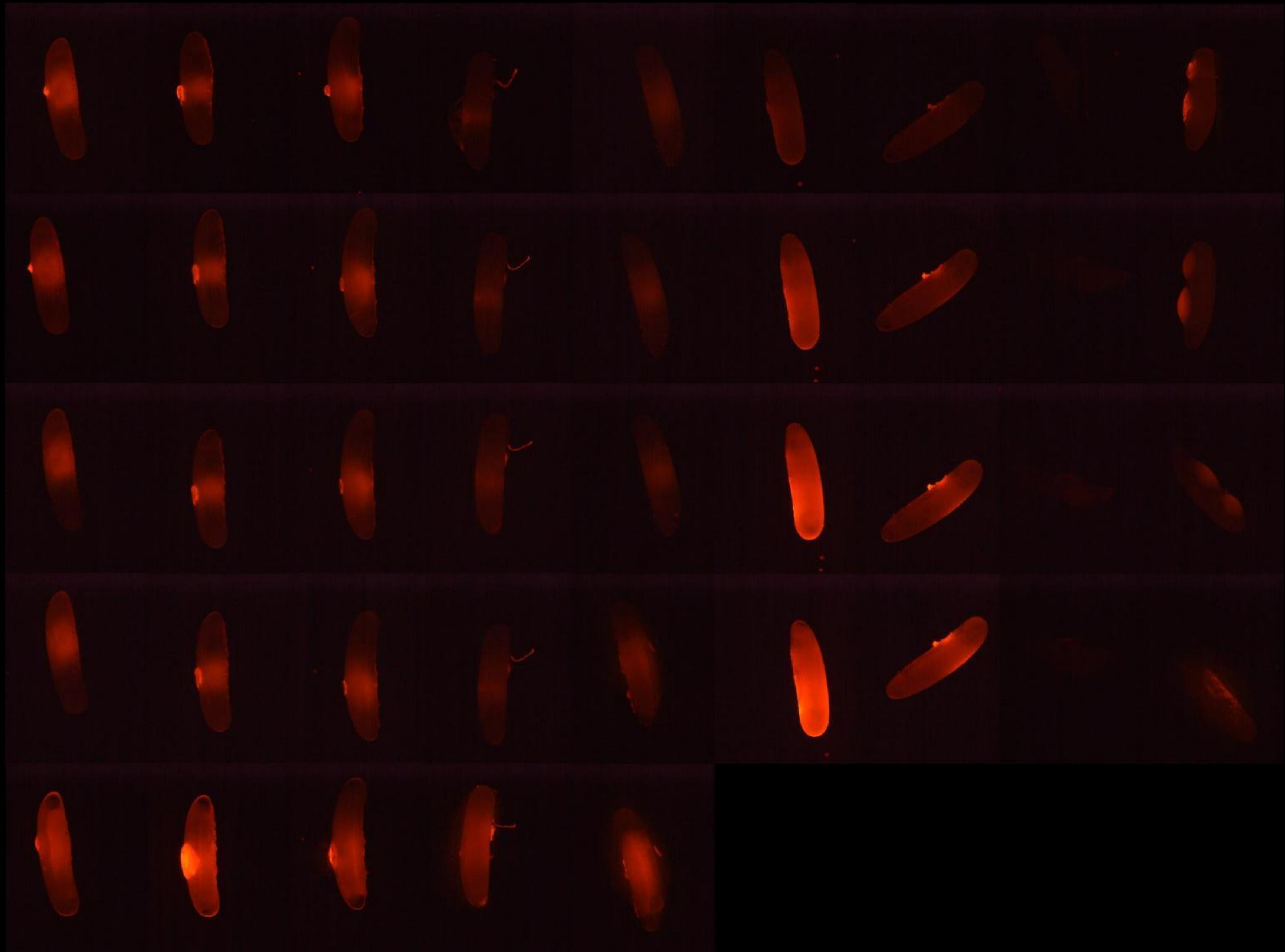

Osmia bicronis – DAR4M-AM injected eggs

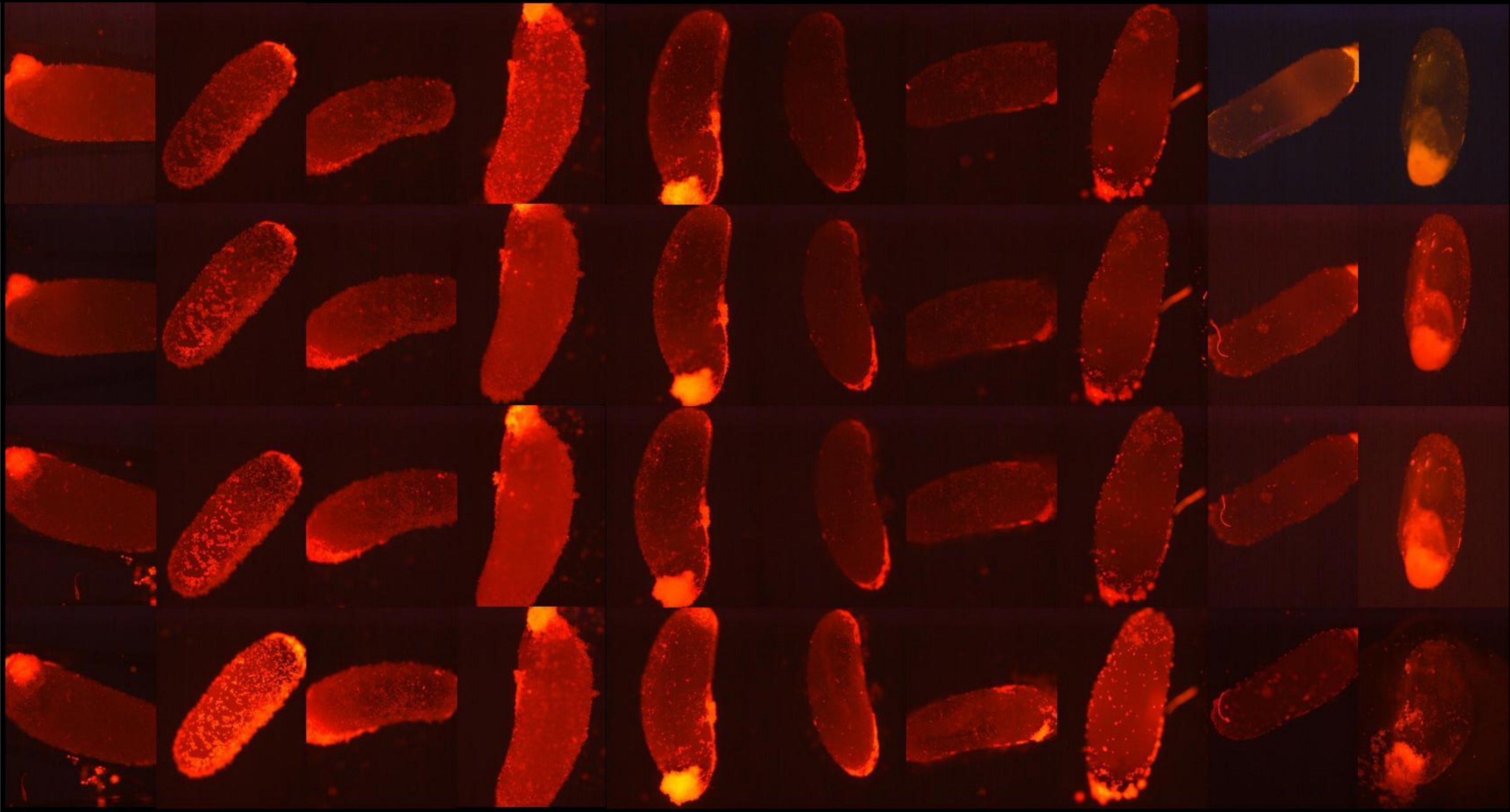

Osmia bicronis – DAR4M-AM injected eggs

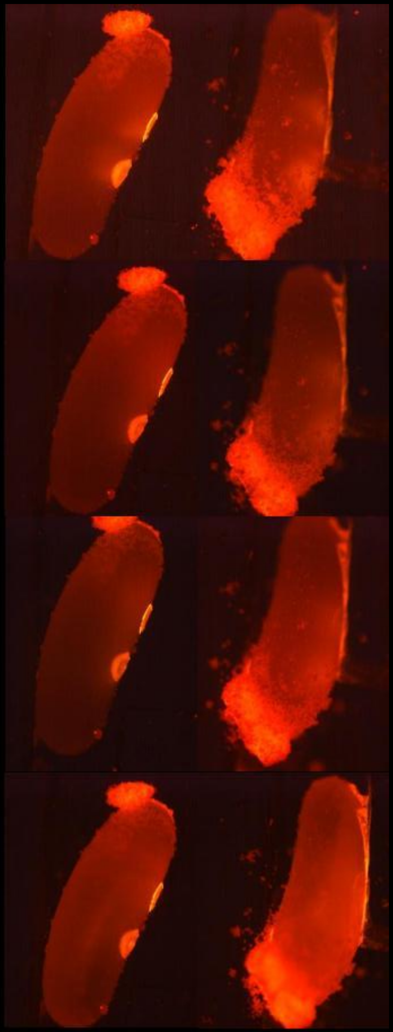

Supplement: Figure 5—source data 1. [file elife-43718-fig5-data1.pdf]
