# Supplementary material for: Nitric oxide radicals are emitted by wasp eggs to kill mold fungi
Source: eLife. 2019 Jun 11;8:e43718. doi: 10.7554/eLife.43718 (PMC6559793; doi:10.7554/eLife.43718)
Supplement: Supplementary file 1. [file elife-43718-supp1.docx]

**Supplementary file 1. Primers used for sequencing of the *Pt-NOS*.**

| **Primer Name** | **Based upon** | **Primer sequence**  **(5’ – 3’)** | **Direction** | **Length** | **Position in**  ***P. triangulum***  **NOS gDNA sequence** |
| --- | --- | --- | --- | --- | --- |
| NOS_qPCR_F2 | *Philanthus triangulum* | CAAATGAAAAACGTAACCATCG | fwd | 22 | 5221-5242 |
| NOS_qPCR_R2 | *P. triangulum* | GTGGTACGATCCACAACCAG | rev | 20 | 5322-5341 |
| Actin_qPCR_F1 | *Apis mellifera, Gryllus bimaculatus, P. triangulum* | GGTAACGAAAGATTCCGTTG | fwd | 20 | - |
| Actin_qPCR_R1 | *A. mellifera, G. bimaculatus, P. triangulum* | GATCCACATCTGTTGGAAGG | rev | 20 | - |
| NOS860fwd2 | *A. mellifera, Drosophila melanogaster, Anopheles stephensi, Rhodnius prolixus, Manduca sexta* | CVTTCARYGGHTGGTAYATG | fwd | 20 | 4973-4992 |
|  |  |  |  |  |  |
|  |  |  |  |  |  |
|  |  |  |  |  |  |
| NOS1571rev1 | *A. mellifera, D. melanogaster, A. stephensi, R. prolixus, M. sexta* | TTTVGAWGTRAAYTTIACWGC | rev | 21 | 5641-5661 |
|  |  |  |  |  |  |
|  |  |  |  |  |  |
|  |  |  |  |  |  |
| NOS_seq_F1_deg | *A. mellifera, Nasonia vitripennis* | ACWGAARCTTTTGATTCTYTGC | fwd | 22 | 198-219 |
| NOS_seq_R1_deg | *A. mellifera, N. vitripennis* | CACCACAWACKTAAAAATGACC | rev | 22 | 8655-8676 |
| NOS_seq_5-F1 | *P. triangulum* | TCACAGGTCACGAGTTCAATCC | fwd | 22 | 919-940 |
| NOS_seq_5-R1 | *P. triangulum* | CCTCTCCAACCCAATTTTATGC | rev | 22 | 4602-4623 |
| NOS_seq_5-F2 | *P. triangulum* | TCTAACTCCTGTAGGACCTAAATTCG | fwd | 26 | 1493-1518 |
| NOS_seq_5-R2 | *P. triangulum* | TTGTGACATAACGGCAATCG | rev | 26 | 4146-4165 |
| NOS_seq_5-F3 | *P. triangulum* | AGCGTGATGCAATTACCAAC | fwd | 20 | 3174-3193 |
| NOS_seq_5-F6 | *P. triangulum* | AATGGAGGAAAACTCAACTGG | fwd | 21 | 20-40 |
| NOS_seq_3-F1 | *P. triangulum* | GAAGTTCCATTTCAAACAAATTGC | fwd | 24 | 5553-5576 |
| NOS_seq_3-R1 | *P. triangulum* | CCGTAATGAACAGGTCCAAATC | rev | 22 | 7988-8009 |
| NOS_seq_3-F2 | *P. triangulum* | GCATTCGGTCGTTACGTG | fws | 18 | 6476-6493 |
| NOS_seq_3-R2 | *P. triangulum* | ACGGCTTAAATTCGTCTTCC | rev | 20 | 7073-7092 |
| NOS_seq_3-F3 | *P. triangulum* | GATTTGGACCTGTCCATTACG | fwd | 21 | 7988-8008 |
| NOS_seq_3-F6 | *P. triangulum* | GATTTGATTCAAGCCGAAGC | fwd | 21 | 8601-8620 |
| NOS_RT_R1 | *P. triangulum* | GCAATCAAACACCATACCAG | rev | 20 | 4864-4883 |
| Adapter+PolyT | *3'RACE, Molecular cloning protocol* | GACTCGAGTCGACATCG  TTTTTTTTTTTTTTTT | rev | 33 |  |
| Adapter | *3'RACE, Molecular cloning protocol* | GACTCGAGTCGACATCG | rev | 17 |  |
| polyT | *Reverse transcription protocol* | TTTTTTTTTTTTTTTTT | - | 17 |  |
