# Supplementary material for: Nitric oxide radicals are emitted by wasp eggs to kill mold fungi
Source: eLife. 2019 Jun 11;8:e43718. doi: 10.7554/eLife.43718 (PMC6559793; doi:10.7554/eLife.43718)
Supplement: Transparent reporting form [file elife-43718-transrepform.pdf]

1st Floor
24 Hills Road
Cambridge CB2 1JP, UK

P 01223 855340
W elifesciences.org
T @elife

eLife's transparent reporting form

We encourage authors to provide detailed information *within their submission* to facilitate the interpretation and replication of experiments. Authors can upload supporting documentation to indicate the use of appropriate reporting guidelines for health-related research (see EQUATOR Network), life science research (see the BioSharing Information Resource), or the ARRIVE guidelines for reporting work involving animal research. Where applicable, authors should refer to any relevant reporting standards documents in this form.

If you have any questions, please consult our Journal Policies and/or contact us: editorial@elifesciences.org.

Sample-size estimation

- You should state whether an appropriate sample size was computed when the study was being designed
- You should state the statistical method of sample size computation and any required assumptions
- If no explicit power analysis was used, you should describe how you decided what sample (replicate) size (number) to use

Please outline where this information can be found within the submission (e.g., sections or figure legends), or explain why this information doesn't apply to your submission:

The requested information on sample sizes is given in the method section under the headline "General experimental procedures".

Since it is rather difficult to obtain beewolf eggs - and in particular at a certain stage - for some experiments, we used as many eggs as possible (e.g. quantitative PCR). For some experiments we used experience from preliminary experiments to decide on a meaningful sample size (e.g. inhibition assays in Petri dishes were really clear-cut and required only few replicates).

Replicates

- You should report how often each experiment was performed
- You should include a definition of biological versus technical replication
- The data obtained should be provided and sufficient information should be provided to indicate the number of independent biological and/or technical replicates
- If you encountered any outliers, you should describe how these were handled
- Criteria for exclusion/inclusion of data should be clearly stated
- High-throughput sequence data should be uploaded before submission, with a private link for reviewers provided (these are available from both GEO and ArrayExpress)

Please outline where this information can be found within the submission (e.g., sections or figure legends), or explain why this information doesn't apply to your submission:

The requested information on replicates is given in the method section under the headline "General experimental procedures".

Information on replicates is given in the Method section. Due to the limited availability of beewolf eggs on a given day, replicates for some experiments were not conducted simultaneously but successively. However, care was taken to keep all procedures and conditions identical. In Figure 6. there is an outlier as indicated by the statistical software, however, we included it in the analysis thus keeping it conservative.

Statistical reporting

- Statistical analysis methods should be described and justified
- Raw data should be presented in figures whenever informative to do so (typically when N per group is less than 10)
- For each experiment, you should identify the statistical tests used, exact values of N, definitions of center, methods of multiple test correction, and dispersion and precision measures (e.g., mean, median, SD, SEM, confidence intervals; and, for the major substantive results, a measure of effect size (e.g., Pearson's r, Cohen's d)
- Report exact p-values wherever possible alongside the summary statistics and 95% confidence intervals. These should be reported for all key questions and not only when the p-value is less than 0.05.

Please outline where this information can be found within the submission (e.g., sections or figure legends), or explain why this information doesn't apply to your submission:

Specification of statistical tests and how they were applied is given in the Method section after the description of the respective experiment.

Survival analysis was used to analyze time event data (timing of fungus growth), hazard ratios are provided in Fig. 2 as a measures of effect size. Binomial tests were used to analyze the outcome of inhibition assays in Petri dishes. Mann Whitney tests with Holm correction were used for group comparisons (NOS inhibition assay).

(For large datasets, or papers with a very large number of statistical tests, you may upload a single table file with tests, Ns, etc., with reference to sections in the manuscript.)

Group allocation

- Indicate how samples were allocated into experimental groups (in the case of clinical studies, please specify allocation to treatment method); if randomization was used, please also state if restricted randomization was applied
- Indicate if masking was used during group allocation, data collection and/or data analysis

Please outline where this information can be found within the submission (e.g., sections or figure legends), or explain why this information doesn't apply to your submission:

The requested information on sample sizes is given in the method section under the headline "General experimental procedures".

Beewolf eggs were randomly assigned to treatment groups.

Additional data files ("source data")

- We encourage you to upload relevant additional data files, such as numerical data that are represented as a graph in a figure, or as a summary table
- Where provided, these should be in the most useful format, and they can be uploaded as "Source data" files linked to a main figure or table
- Include model definition files including the full list of parameters used
- Include code used for data analysis (e.g., R, MatLab)
- Avoid stating that data files are "available upon request"

Please indicate the figures or tables for which source data files have been provided:

Fig 2 Source data – effect of egg on fungus growth.xlsx
Fig 5 Source data - timing of NO emission.xlsx
Fig 6 source data – NOS inhibition.xlsx
Fig S2 Source data - start of NO emission.xlsx
Fig S4 Source data - NOS gene expression.xlsx
